# Genetic Sensitivity to the Bitter Taste of 6-*n*-Propylthiouracil (PROP) and Its Association with Physiological Mechanisms Controlling Body Mass Index (BMI)

**DOI:** 10.3390/nu6093363

**Published:** 2014-08-27

**Authors:** Beverly J. Tepper, Sebastiano Banni, Melania Melis, Roberto Crnjar, Iole Tomassini Barbarossa

**Affiliations:** 1 Department of Food Science, School of Environmental and Biological Sciences, Rutgers University, New Brunswick, NJ 08901-8520, USA; E-Mail: tepper@AESOP.Rutgers.edu; 2 Department of Biomedical Sciences, University of Cagliari, Monserrato, CA 09042, Italy; E-Mails: banni@unica.it (S.B.); melaniamelis@unica.it (M.M.); crnjar@unica.it (R.C.)

**Keywords:** PROP taste phenotype, BMI, endocannabinoid system

## Abstract

Taste sensitivity to the bitter compound 6-*n*-propylthiouracil (PROP) is considered a marker for individual differences in taste perception that may influence food preferences and eating behavior, and thereby energy metabolism. This review describes genetic factors that may contribute to PROP sensitivity including: (1) the variants of the TAS2R38 bitter receptor with their different affinities for the stimulus; (2) the gene that controls the gustin protein that acts as a salivary trophic factor for fungiform taste papillae; and (3) other specific salivary proteins that could be involved in facilitating the binding of the PROP molecule with its receptor. In addition, we speculate on the influence of taste sensitivity on energy metabolism, possibly via modulation of the endocannabinoid system, and its possible role in regulating body composition homeostasis.

## 1. Introduction

Taste is the sensory modality that enables organisms to distinguish nutrient-rich food from noxious substances, and acts as a final checkpoint for food acceptance or rejection behavior [1,2]. In humans, it is generally assumed that the taste system can differentiate five primary sensory qualities (sweet, umami, sour, salty, and bitter). These taste qualities act synergistically to orchestrate appetitive responses to energy- and protein-rich food sources (sweet, fatty acids and umami), govern intake of an adequate amount of sodium (low-salt taste), and warn against the ingestion of toxic substances or excess salt (bitter, sour and high-salt tastes) [2]. In addition, the ability to taste fatty acids has been recently proposed as a sixth primary sensory quality [3] and has been confirmed by different groups [4,5,6]. Interestingly, fatty acids are detected by the plasma membrane lipid-binding protein CD36, which plays a crucial role in the oro-sensory perception of dietary lipids in mammals [6,7]. Indeed, CD36 gene disruption has been shown to abolish fat preference and intake in the mouse [8,9]. In addition, humans seem to discriminate among fatty acids, probably based on the presence of double bonds, and genetic variation in taste sensitivity to PROP seems to affect chemosensory responses to unsaturated fatty acids [4].

Taste sensitivity varies greatly among individuals and may be one of the most important determinants influencing food choice and therefore the nutritional status and health of the individual [10]. It is well known to even the casual observer that the same kind of food can taste very different to two individuals. This difference depends, in part, on cultural and social factors, but there is also an important genetic component. The genetic component of taste variability could be the result of evolutionary adaptation mechanisms to specific environments to recognize substances potentially harmful or necessary for bodily functions [11]. For example, since many bitter-tasting substances can be toxic, the ability of humans to detect bitterness at low concentrations represents an evolutionary adaptation for limiting their consumption [12]. On the other hand, several classes of bitter polyphenols, such as those found in tea, coffee, dark-colored fruit, citrus, and chocolate, provide positive health benefits, so low sensitivity encourages their consumption [13,14].

Whether taste sensitivity plays a role in controlling the metabolism of ingested nutrients is still debated. It is also unclear under which mechanisms taste sensitivity may influence macronutrient intake. Importantly, foods are consumed as macromolecules (e.g., proteins, starches, triglycerides) not as isolated components. However, the gustatory system is configured to respond to single units and breakdown products such as amino acids, free fatty acids, and mono- and di-saccharides. With the exception of the saccharides, these components are not abundant in human diets. Digestive enzymes such as amylase and lipases are present in saliva that degrade macronutrients into these single units [15]. These molecules can then interact through a variety of oral sensing mechanisms to convey signals about the quantity and quality of the ingested nutrients contributing to the efficient metabolism and disposal of such nutrients. It would also be crucial for this system to respond to danger by sensing the presence of excess free fatty acids or amino acids that may indicate the presence of food degradation or contamination by hydrolytic microorganisms. Understanding the range of oral sensibilities in human beings and how it is influenced by genetic and environmental variables may lead to important insights about the role of taste in food intake regulation and metabolism.

## 2. Physiological Overview of Taste Sensitivity

Taste in humans begins with the activation of the epithelial-derived taste cells where taste reception and signal transduction mechanisms are located. Groups of taste cells (50 to 100) are organized into taste buds situated on the surface of papillae. There are three different functional taste papillae types that are topographically arranged mostly on the superior surface of the tongue. Fungiform papillae are found in the anterior two-thirds of the tongue, foliate papillae on the lateral sides, and circumvallate papillae on the posterior one-thirds.

Taste buds show an elegant functional organization in which different cell types with opposing effects of positive and negative feedback are integrated to shape the neural output transmitted to the hindbrain [2]. Bud cells are distinguished into functional classes by combined analyses of gene expression and cellular function. Overall, Type I cells appear to function as glia in taste buds, though they may exhibit ionic currents implicated in salt taste transduction [16]. Type II cells are “receptor” cells for the transduction of sweet, bitter, and umami taste stimuli. G protein-coupled receptors (GPCRs) localized to the plasma membranes of Type II cells bind sweet, bitter, or umami compounds. Each GPCR class is expressed in its own distinct taste cell type, which responds to ligands that bind those specific receptors [17]. This one-taste/one-cell-class coding scheme is a mechanism through which taste qualities are detected and codified on the tongue [18,19]. Sweet and umami transduction are mediated by a small family of heterodimer GPCRs: T1R2 + T1R3 for sweet-tasting compounds [17,20,21]; and T1R1 + T1R3 for umami [22,23]. Some authors have also suggested that other candidate receptors for sweet and umami may exist [24,25,26]. Finally, bitter taste is mediated by a large family of GPCRs known as T2R receptors. Humans possess *ca.* 25 T2Rs encoded by clusters of genes located on chromosomes 5p, 7q, and 12p [27]. T2Rs respond to a diversity of bitter taste molecules [28,29,30,31,32], but they exhibit different ranges of specificity: some are a highly-selective, responding to a limited number of compounds, while other are highly promiscuous, responding to numerous bitter compounds [32]. Type II cells also express voltage-gated Na^+^ and K^+ ^channels as well as hemichannels (Panx1) involved in the generation of action potentials and in the taste-induced ATP secretion to excite specific ATP receptors in nervous fiber and taste cells. These “receptors cells” do not form specialized synapses with taste sensory nerve fibers, which exist in close proximity to their basal pole. Type III cells are labeled “presynaptic cells” as they form synaptic junctions with gustatory nerve terminals [33,34,35,36]. “Presynaptic cells” release at least two neurotransmitters, serotonin (5-HT) and norepinephrine (NE). Type III cells respond directly to sour taste stimuli and carbonated (CO_2_) solutions by way of ion channels [37,38,39,40] but, they can also integrate signals that they receive from Type II cells. Thus, Type III cells are not specific for a given sensory quality, but instead respond to compounds of all qualities. Finally, a class of nonpolarized and undifferentiated cells termed “basal cells” are also present in taste buds.

Stimulants evoke a series of chemical signals that are integrated in the taste bud before taste information is transmitted to gustatory nerve fibers. The activation of Type II cells by sweet, bitter or umami stimuli induces secretion of ATP through Panx1 hemichannels. The extracellular ATP exerts three different functions mediated by ATP receptors (P2X, P2Y): activation of gustatory afferent nerve fibers; activation of adjacent presynaptic cells which release 5-HT and/or NE; and autocrine signaling via a positive feedback mechanism onto receptor cells that increases their own secretion.

Taste signals integrated in taste buds are transmitted by fibers of the three cranial nerves (VII, IX and X) to the rostral portion of the solitary tract nucleus (NST) of the medulla. This information is transferred to the thalamus (ventral posteromedial nucleus), and hence to the gustatory areas of the cortex in the insula where it gives rise to the taste sensation. Local projections from the NST within the brainstem mediate non-cortical behavioral responses, such as those related to food ingestion or rejection. Gustatory signals from the NST also project to feeding centers in the amygdala and the hypothalamus where they can modulate hunger and fullness.

How the taste signals integrated within taste buds are translated into a neural code for the perception of different taste qualities remains an open question. Three theories have been widely discussed: the labeled line (LL) theory which states that dedicated fibers transmit each sensory quality; the across fiber pattern (AFP) theory which posits that qualities are encoded by patterns of activity across several fibers; and finally a theory of temporal coding. According to the temporal coding theory, taste qualities are deciphered by different frequencies and/or timing patterns of action potential discharges [41,42]. Although, researchers in the field agree that labeled taste lines exist [2], it is certain that, like cells in taste buds, some fibers respond strongly to a single taste quality, while others are responsive to multiple taste qualities.

The minimal gustatory circuitry and basic taste models described above are not sufficient to explain complex behavioral taste-induced processes. Moreover, the taste network in the brain is too extensive (over 20 brain regions are implicated in taste processing) and heavily interconnected (over 40 connections) via reciprocal pathways, to be adequately described by traditional feed-forward models of taste coding [43]. The internal dynamics of this extensive neural network have profound effects on gustatory perception and behavior, and must be considered to effectively link taste detection, and food preferences, with appetite regulation.

## 3. Genetic Factors Contributing to PROP Sensitivity

The genetic basis of taste variability was accidentally discovered by Arthur L Fox in 1931, while he was working in his laboratory to synthesize non-nutritive sweeteners. Fox found that people varied in their response to the bitter synthetic compound phenylthiocarbamide (PTC). Subsequent tests showed that about 30% of individuals could not taste PTC (non-tasters), whereas the majority could taste it as moderately or intensely bitter (tasters) [44]. These same findings have also been reported for PROP which is chemically similar to PTC [45]. By using suprathreshold screening methods, Bartoshuk and co-authors first identified a subgroup of tasters, named super-tasters, who were very sensitive to PROP/PTC [46,47]. The frequency of non-tasters varies greatly among populations around the globe (from as low as 7% to more than 40%) and depends on race and ethnicity [48].

Some studies have consistently reported that individuals who differ in their taste response to PROP/PTC are also anatomically different. In particular, there is considerable evidence that super-tasters have a greater density of fungiform taste papillae on the anterior surface of the tongue, when compared to the other PROP taster groups [47,49,50,51,52,53,54].

**Figure 1:**
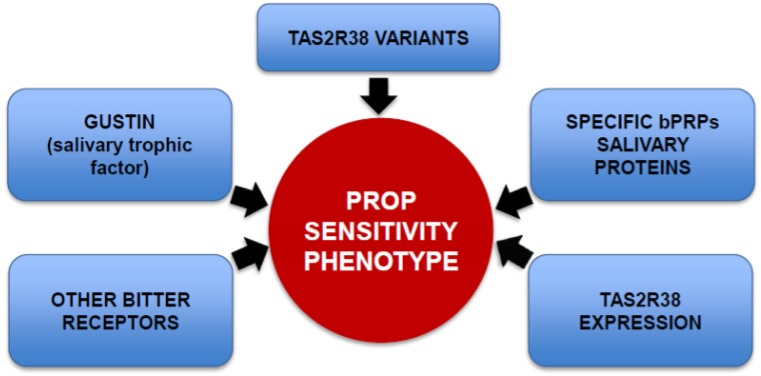
Graphic diagram representing the principal genetic factors that contribute to PROP taste sensitivity phenotype. These factors include: the salivary trophic factor gustin, a protein that provides the mechanistic explanation for why PROP super-tasters are more responsive to stimuli that are not mediated via the TAS2R38 bitter receptor; TAS2R38 variants with their different affinities for the PROP stimulus; specific salivary peptides belonging to the basic proline-rich protein family (bPRP), which could facilitate binding of PROP with its receptor site; the involvement of other bitter receptors which may be associated with supertasting and PROP bitterness; and greater mRNA expression associated with the PAV allele of the TAS2R38 receptor which correlates with greater PROP bitterness perception.

The ability to taste PROP is a heritable trait [55]. The gene most closely associated with PROP phenotype variance is TAS2R38 that expresses receptors that bind the N–C=S group responsible for the bitter taste of thiourea compounds [56,57]. The allelic diversity in this gene is due to three single-nucleotide polymorphisms (SNPs) which result in three amino acid substitutions (Pro49Ala, Ala262Val, and Val296Ile) and give rise to two common haplotypes: PAV, the dominant taster variant and AVI, the non-taster recessive one. Also, rare haplotypes (AAV, AAI, and PVI) have been observed to contribute to intermediate PROP/PTC sensitivity [56,57]. Haplotypes of this gene do not completely explain phenotypic differences in PROP tasting, especially between medium tasters and super-tasters. This discrepancy implies that other factors may be involved in the expression of this complex trait, in addition to the TAS2R38 variants and their different affinities for the stimulus (Figure 1). For example, Hayes and co-workers [58] suggested that other bitter receptors may be involved in tasting PROP, especially at high concentrations, and could explain the misclassification of some AVI homozygous individuals in their study. In another study, Lipcock* et al.* showed that PROP bitterness intensity was strongly associated with mRNA expression of the PAV-TAS2R38 allele, an (indirect) index of receptor protein production [59]. Finally, other studies using a variety of approaches have suggested that modifying genes may also play a role in the ability to taste PTC/PROP [60,61,62].

Individual differences in PROP bitterness have also been attributed to many other factors including the chemical composition of saliva, its physical properties and the number, size and morphology of taste papillae. Taste stimuli, in the mouth, must diffuse through the salivary fluid layer to penetrate the taste pore. Once they gain access to the taste pore cavity, taste molecules interact with receptor sites on the plasma membrane of microvilli, which extend from the apical portion of taste cells [63]. This process includes the solubilization of chemicals in saliva and their interaction with salivary components. In this respect, saliva is a key element of the initial processes of taste transduction, and its chemical-physical variation can affect taste sensitivity, as well as the health and integrity of the taste cells [63].

As early as the first half of the twentieth century, Fox [44] suggested that the interaction between salivary chemical constituents and taste stimuli could explain the large phenotypic differences in the bitter taste perception of thiourea compounds. Fox speculated that the taste blindness of non-tasters may be due to the presence in saliva of products (such as proteins or colloids) which precipitate taste substances inhibiting their perception. Since that time, a large body of literature has focused on the ability of salivary proteins, such as the Proline Rich Proteins (PRPs), to bind and precipitate plant polyphenols in the oral cavity during astringency perception [64,65,66,67]. A recent study has focused on the salivary proteome as an additional layer of genetic diversity that contributes to individual differences in the PROP bitterness perception [68]. In this study, PROP responsiveness was directly associated with levels of two salivary peptides belonging to the basic proline-rich protein family (bPRP), namely Ps-1 and II-2, which derive from the cleavage of pro-proteins expressed by S, M, and L alleles of the PRB1 gene [69]. Since the Ps-1 protein only derives from the M allele of this gene, Cabras and colleagues [68] speculated that PROP super-tasting, which was related to high Ps-1 levels, might also be associated with the M allele of this gene.

The functional meaning of these proteins in saliva and the physiological mechanisms by which they facilitate the perception of PROP bitterness have been further investigated by Melis* et al.* [70]. These authors showed that oral supplementation with Ps-1 protein in subjects lacking it in saliva markedly increased their PROP bitter taste responsiveness, and the effect was more potent in non-tasters than in the other PROP taster groups. In addition, these studies highlighted the importance of constituent amino acids (l-Arg and l-Lys) that selectively interact with the PROP molecule by facilitating its binding with the TAS2R38 taste receptor.

The multiplicity of genetic and environmental factors that appear to influence PROP sensitivity, as well as the inherent genetic diversity in this trait across populations, make it difficult to identify individual, relevant factors that contribute to PROP tasting. In this regard, the study of genetically homogeneous populations are valuable since they tend to minimize background noise associated with the characterization complex traits. We studied a homogenous genetic cohort on the island of Sardinia and showed that a key factor strongly associated with PROP taste sensitivity is the polymorphism, rs2274333 (A/G), located in the gustin (CA6) gene that controls the zinc-dependent salivary protein of the same name [53,71]. Gustin protein was previously described as a trophic factor for taste buds [72]. This polymorphism results in the amino acid substitution at position Ser90Gly in the gustin protein sequence. In the Sardinian population, PROP super-tasters more frequently were homozygous for the A allele and expressed the more active enzyme iso-form, whereas non-tasters more frequently carried the GG genotype and expressed the less functional form of the protein [71]. Individuals with the GG genotype also had a lower density of fungiform papillae and exhibited a higher proportion of unusually large and distorted fungiform papillae, than did subjects with the more functional allele, suggesting an association of the gustin gene with growth and maintenance of taste papillae [53]. Moreover, Melis and co-workers [53] showed, in* in vitro* experiments, that isolated cells thrive better when exposed to saliva from AA subjects or the corresponding active iso-form (Ser90) of the protein, thus reinforcing the association between the gustin gene and the formation and function of papillae. Although gustin and TAS2R38 have been shown to have independent effects on PROP tasting, together they account for up to 60% of the variance in PROP bitterness intensity. In contrast, only 40% of the variance in PROP taste threshold is due to the combined effects of gustin and TAS2R38 [73]. In addition, the contribution of the gustin gene in each TAS2R38 genotype group showed that a single A allele was sufficient for individuals to exhibit decreased thresholds while two alleles (AA) were needed to determine increased bitterness intensity. These data suggest that the PAV variant receptor is more important for perceiving high concentrations of PROP, while the gustin gene is more relevant for detecting low concentrations. The role of gustin remains controversial, however, since some studies have shown no relationship between gustin polymorphisms and PROP sensitivity or papillae density in genetically-diverse cohorts. Specifically, Genick and co-authors [74] found no relationship between gustin and PROP phenotypes in a genome-wide association study conducted in Brazil. Likewise, Feeney and Hayes [75] failed to find evidence that CA6 affects PROP taste perception by modifying fungiform papillae density in a genetically mixed cohort in the United States.

Controversial data exist on the involvement of gender in individual differences in PROP perception. Some studies showed that women are more frequently tasters compared with men [47,76,77]. Women are also more likely to be super-tasters, [47] and to have more taste buds and fungiform papillae. However, other authors did not report these same results [53,78,79].

## 4. Nutritional Implications of PROP Bitter Taste Sensitivity

Several studies in human nutrition have suggested that the PROP phenotype may serve as a general marker for oral sensations and food preferences, thus influencing dietary behavior and nutritional status [10]. It has also been reported that PROP super-tasters have a higher sensitivity than non-tasters to various oral stimuli, including other bitter-tasting compounds and foods such as dark chocolate, black coffee, caffeine solutions, soy products and green tea [80], sweet substances, chemical irritants (chili or ethanol), and the texture of fats [46,50,54,81,82,83,84,85,86,87,88,89,90]. Other reports show that those individuals who perceive PROP as extremely bitter typically show a lower acceptance of *Brassica* vegetables, and also avoid strong-tasting versions of foods that do not contain the thiourea groups including sweets, spicy foods and alcoholic beverages [10,54,90,91,92,93,94,95,96]. Given the nutritional importance of dietary lipids, the relationship between PROP status and perception and liking of fat have been extensively investigated. Most studies [54,90,97,98,99,100] but not all [101,102], reported that PROP non-tasters had a lower ability to distinguish fat content and creaminess in certain fatty foods. In particular, Tepper and Nurse [54] showed that non-tasters could not discriminate a high-fat from a low-fat salad dressing, whereas tasters reliably distinguished the two samples. Moreover, PROP non-tasters showed higher preferences for dietary fat (such as full-fat milk, high-fat salad dressings and sweet-fat dairy mixtures) [90,92,93,99,103,104] and consumed more servings of discretionary fats and high-energy foods per day than did tasters [93,105]. Finally, PROP tasters gave higher taste intensity ratings for linoleic acid, an essential polyunsaturated fatty acid, compared with PROP non-tasters [4].

These findings support the hypothesis of an inverse correlation between PROP tasting and calorie consumption and/or BMI, which has been reported in several studies [76,105,106,107,108]. However, other reports have produced conflicting evidence suggesting that other factors may play a role in defining the pathway linking PROP tasting and food perception and preference, with feeding behaviour and body weight [102,103,109,110,111,112,113].

## 5. Variables that May Influence the Relationship between PROP Sensitivity and BMI

**Figure 2:**
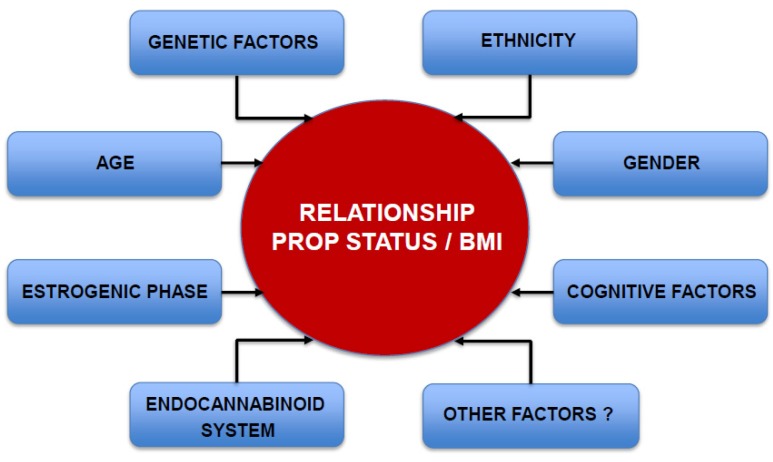
Graphic diagram showing variables so far identified that influence the relationship between PROP taste sensitivity and BMI.

Several studies have focused on identifying the factors that may lead to divergent conclusions about the involvement of the PROP phenotype in food preferences, dietary choice and BMI (Figure 2). One of the major issues is the difficulty in obtaining an objective measure of a subject’s chemosensory phenotype. This could be due, in part, to the lack of universally-accepted psychophysical testing methods as well as the inability to directly measure the degree of gustatory system activation in humans. Psychophysical approaches include threshold measures to determine the lowest stimulus concentration which can be distinguished from reference samples, and suprathreshold methods that utilize rating scales to assess responsiveness at higher concentrations [10,47,74,114,115]. Suprathreshold methods are highly subjective because individuals utilize scales based on their personal experiences [74]. Genick and co-authors estimated that measurement errors account for 20% of PROP phenotypic variance [74]. However, both kinds of psychophysics approaches showed high intra-subject variability not attributable to measurement errors [74] that are comparable with changes observed by others [114,116,117].

The role of hormonal fluctuations due to the menstrual cycle on taste sensitivity is conflicting [118]. Variation in taste perception due to the estrogenic phase has been reported [119,120,121], suggesting that it should be taken into consideration when testing fertile women. However other authors find only minimal changes [122]. Several studies suggest that taste sensitivity diminishes with increasing age [84,123,124,125]. The specific effect of age has been shown on PROP bitterness in individuals heterozygous for the PAV/AVI diplotype, with children being more responsive to PROP than adults [116]. Age was also associated with modestly higher PROP thresholds, accounting for 5%–8% of the variance in taste acuity [53,74,77,123]. Although the frequency of non-tasters varies greatly among populations around the globe [48], Genick and co-authors found that demographic parameters, such as BMI or ethnicity, had only a very small influence on PROP detection threshold [74].

It is known that the cognitive control of eating behavior plays an important role in determining the relationship between PROP phenotype and BMI, especially in women [71,106]. Since dietary restraint (conscious control of eating) [126] of food intake is often associated with a lower energy intake, decreased fat intake [127,128] and a more frequent use of products with low fat content [129,130], it is not surprising that restrained subjects are less influenced by taste in choosing foods and more influenced by the concern to maintain an acceptable BMI [106]. Accordingly, Tepper and Ullrich [106] observed that non-taster women with low dietary restraint showed the expected negative association between PROP status and BMI, but this association was masked in women who voluntarily restrained their food intake. A second characteristic of eating behavior is disinhibition, that is defined as a loss of control over eating in response to various types of stress and negative emotional states (such as anxiety, anger), [126]. Disinhibition is strongly associated with disruptions in eating behavior [130,131], increased adiposity [106] and higher intake of appetizing energy dense foods, which contributes to overweight/obesity [132]. Although Tepper and Ullrich [106] showed that disinhibition had a strong independent effect on BMI, it did not influence the relationship between PROP status and body weight in their study. In contrast, Tomassini Barbarossa* et al.* [133] showed that PROP non-taster individuals had disinhibition scores that were almost two-fold higher than those of super-tasters. Together, these findings suggest that cognitive eating behaviors may vary considerably across different subject populations and could exert variable effects on the association between PROP and weight status.

**Figure 3:**
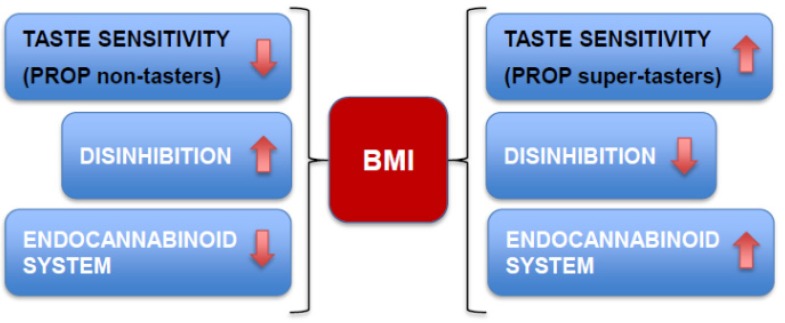
Graphic diagram showing a possible mechanism describing the interactions among the endocannabinoid system, disinhibition (loss of control over eating) and PROP taste sensitivity in the control of body weight. According to this mechanism, lower plasma levels of endocannabinoids may counteract overeating in disinhibited, non-tasters, restoring food intake and body weight to normal limits. In super-tasters with low disinhibition, higher levels of endocannabinoids may be associated with normal regulation of food intake and body weight. This mechanism may explain why some non-tasters maintain normal BMIs despite the tendency for hedonic overeating in such individuals.

Factors that influence energy metabolism may also affect the relationship between PROP status and BMI. One example is the endocannabinoid system that may work to fine-tune body metabolism in response to dietary exposure to taste stimuli. The endocannabinoid system regulates “on demand” production and degradation, by specific pathways, of arachidonic acid derivatives, *N*-arachidonoylethanolamide (anandamide, AEA) and 2-arachidonoylglycerol (2-AG), and their high affinity cannabinoid receptors (CB) 1 and CB 2 [134]. These receptors are widely expressed in peripheral tissues and the central nervous system. The endocannabinoid system has been shown to play a crucial role in energy metabolism by influencing food intake and reward at the level of the hypothalamus and nucleus accumbens respectively, as well as by modulating energy expenditure in peripheral tissues in experimental models and humans, as described in a recent and comprehensive review [134]. Dietary fatty acids can modulate circulating endocannabinoid levels [135], by affecting tissue levels of arachidonic acid, the precursor of the endocannabinoids, as demonstrated in mice [136]. Moreover, it has been recently shown that the endocannabinoid system influences dietary fat sensitivity in both the oral cavity and intestine via CB1 receptors [137]; endocannabinoids also enhance hedonic eating [138]. These data suggest that, the endocannabinoid system may regulate body energy storage and metabolism, based on energy needs and genetic factors that influence taste sensitivity. To test this hypothesis, we recently investigated whether PROP sensitivity, through its influence on eating behavior, also modifies endocannabinoid biosynthesis [133]. Interestingly, we found that normal weight non-tasters compared to normal weight super-tasters, had lower circulating levels of both AEA and 2-AG. We suggest that lower levels of circulating endocannabinoids may counteract the tendency of non-tasters to overeat as a consequence of their higher disinhibition which was also observed in this study (Figure 3). Thus, the differences in endocannabinoid levels between super-tasters and non-tasters may represent a mechanism to regulate energy intake and normalize impaired feeding behavior.

Finally, many bioactive compounds that are regularly present in very low amounts in our diet, or added to foods as spices, may influence energy balance. For example, terpenes and polyphenols, with strong taste and flavor components, possess sympathomimetic properties that increase thermogenesis and boost energy metabolism [139]. Capsaicin, the compound responsible for the burn of chili peppers via the TRPV1 receptor, may contribute to body weight management, most likely through activation of the sympathetic nervous system [140]. This mechanism is supported by evidence showing that impaired sympathoadrenal activity contributes to obesity [141]. PROP non-tasters are less sensitive to the sensory properties of these compounds and are more likely to consume them. Thus, non-tasters may benefit more from the energy enhancing effects of these compounds than super-tasters who consume these compounds less frequently.

## 6. Conclusions

Recent discoveries elucidating the role of PROP and other taste phenotypes, such as gustin, in body weight provide critical insights for understanding the influence of taste sensitivity on eating behavior, energy metabolism, BMI and health. Gustin may be of particular importance because of its role as a trophic factor for taste bud density and maintenance. Future studies should be aimed at evaluating genetic, metabolic and anthropometric variables that interfere with the relationship between taste sensitivity and BMI in diverse physiological or pathological conditions that may induce substantial changes in BMI.

While genetic factors are not modifiable by dynamic environmental changes, other factors, such as the endocannabinoid system is responsive to diet changes, and may function to fine-tune body metabolism according to nutritional content. A more comprehensive approach to weight management should consider the influence of dietary fats on the endocannabinoid system as well as the role of bioactive flavor compounds in energy metabolism. Thus, better characterization of the factors that modify these systems would help us to target populations at-risk and to design diets with tailor-made supplements and/or functional foods, to optimize health.

## References

[1] Scott K. (2005). Taste recognition: Food for thought. Neuron.

[2] Chaudhari N., Roper S.D. (2010). The cell biology of taste. J. Cell Biol..

[3] Mattes R.D., Montmayeur J.P., le Coutre J. (2010). Fat taste in humans: Is it a primary?. Fat Detection: Taste, Texture, and Post Ingestive Effects.

[4] Ebba S., Abarintos R.A., Kim D.G., Tiyouh M., Stull J.C., Movalia A., Smutzer G. (2012). The examination of fatty acid taste with edible strips. Physiol. Behav..

[5] Mattes R.D. (2011). Oral fatty acid signaling and intestinal lipid processing: Support and supposition. Physiol. Behav..

[6] Pepino M.Y., Love-Gregory L., Klein S., Abumrad N.A. (2012). The fatty acid translocase gene CD36 and lingual lipase influence oral sensitivity to fat in obese subjects. J. Lipid Res..

[7] Martin C., Chevrot M., Poirier H., Passilly-Degrace P., Niot I., Besnard P. (2011). CD36 as a lipid sensor. Physiol. Behav..

[8] Laugerette F., Passilly-Degrace P., Patris B., Niot I., Febbraio M., Montmayeur J.P., Besnard P. (2005). CD36 involvement in orosensory detection of dietary lipids, spontaneous fat preference, and digestive secretions. J. Clin. Invest..

[9] Sclafani A., Ackroff K., Abumrad N.A. (2007). CD36 gene deletion reduces fat preference and intake but not post-oral fat conditioning in mice. Am. J. Physiol. Regul. Integr. Comp. Physiol..

[10] Tepper B.J. (2008). Nutritional implications of genetic taste variation: The role of PROP sensitivity and other taste phenotypes. Annu. Rev. Nutr..

[11] Soranzo N., Bufe B., Sabeti P.C., Wilson J.F., Weale M.E., Marguerie R., Meyerhof W., Goldstein D.B. (2005). Positive selection on a high-sensitivity allele of the human bitter-taste receptor TAS2R16. Curr. Biol..

[12] Wooding S., Kim U.K., Bamshad M.J., Larsen J., Jorde L.B., Drayna D. (2004). Natural selection and molecular evolution in PTC, a bitter-taste receptor gene. Am. J. Hum. Genet..

[13] Drewnowski A., Gomez-Carneros C. (2000). Bitter taste, phytonutrients, and the consumer: A review. Am. J. Clin. Nutr..

[14] D’Archivio M., Filesi C., di Benedetto R., Gargiulo R., Giovannini C., Masella R. (2007). Polyphenols, dietary sources and bioavailability. Ann. Ist. Super. Sanita.

[15] Mandel A.L., Breslin P.A.S. (2012). High endogenous salivary amylase activity is associated with improved glycemic homeostasis following starch ingestion in adults. J. Nutr..

[16] Vandenbeuch A., Clapp T.R., Kinnamon S.C. (2008). Amiloride-sensitive channels in type I fungiform taste cells in mouse. BMC Neurosci..

[17] Nelson G., Hoon M.A., Chandrashekar J., Zhang Y., Ryba N.J., Zuker C.S. (2001). Mammalian sweet taste receptors. Cell.

[18] Yarmolinsky D.A., Zuker C.S., Ryba N.J. (2009). Common sense about taste: From mammals to insects. Cell.

[19] Chandrashekar J., Hoon M.A., Ryba N.J., Zuker C.S. (2006). The receptors and cells for mammalian taste. Nature.

[20] Jiang P., Ji Q., Liu Z., Snyder L.A., Benard L.M., Margolskee R.F., Max M. (2004). The cysteine-rich region of T1R3 determines responses to intensely sweet proteins. J. Biol. Chem..

[21] Xu H., Staszewski L., Tang H., Adler E., Zoller M., Li X. (2004). Different functional roles of T1R subunits in the heteromeric taste receptors. Proc. Natl. Acad. Sci. USA.

[22] Li X., Staszewski L., Xu H., Durick K., Zoller M., Adler E. (2002). Human receptors for sweet and umami taste. Proc. Natl. Acad. Sci. USA.

[23] Nelson G., Chandrashekar J., Hoon M.A., Feng L., Zhao G., Ryba N.J., Zuker C.S. (2002). An amino-acid taste receptor. Nature.

[24] Damak S., Rong M., Yasumatsu K., Kokrashvili Z., Varadarajan V., Zou S., Jiang P., Ninomiya Y., Margolskee R.F. (2003). Detection of sweet and umami taste in the absence of taste receptor T1R3. Science.

[25] Maruyama Y., Pereira E., Margolskee R.F., Chaudhari N., Roper S.D. (2006). Umami responses in mouse taste cells indicate more than one receptor. J. Neurosci..

[26] Yasumatsu K., Horio N., Murata Y., Shirosaki S., Ohkuri T., Yoshida R., Ninomiya Y. (2009). Multiple receptors underlie glutamate taste responses in mice. Am. J. Clin. Nutr..

[27] Shi P., Zhang J., Yang H., Zhang Y.P. (2003). Adaptive diversification of bitter taste receptor genes in mammalian evolution. Mol. Biol. Evol..

[28] Chandrashekar J., Mueller K.L., Hoon M.A., Adler E., Feng L., Guo W., Zuker C.S., Ryba N.J. (2000). T2Rs function as bitter taste receptors. Cell.

[29] Roper S.D. (2007). Signal transduction and information processing in mammalian taste buds. Pflugers Arch..

[30] Behrens M., Reichling C., Batram C., Brockhoff A., Meyerhof W. (2009). Bitter taste receptors and their cells. Ann. N. Y. Acad. Sci..

[31] Mueller K.L., Hoon M.A., Erlenbach I., Chandrashekar J., Zuker C.S., Ryba N.J. (2005). The receptors and coding logic for bitter taste. Nature.

[32] Meyerhof W., Batram C., Kuhn C., Brockhoff A., Chudoba E., Bufe B., Appendino G., Behrens M. (2010). The molecular receptive ranges of human TAS2R bitter taste receptors. Chem. Senses.

[33] Murray R.G. (1993). Cellular relations in mouse circumvallate taste buds. Microsc. Res. Tech..

[34] Murray R.G., Murray A., Fujimoto S. (1969). Fine structure of gustatory cells in rabbit taste buds. J. Ultrastruct. Res..

[35] Yang R., Crowley H.H., Rock M.E., Kinnamon J.C. (2000). Taste cells with synapses in rat circumvallate papillae display snap-25-like immunoreactivity. J. Comp. Neurol..

[36] Ye Q., Heck G.L., DeSimone J.A. (1991). The anion paradox in sodium taste reception: Resolution by voltage-clamp studies. Science.

[37] Chandrashekar J., Yarmolinsky D., von Buchholtz L., Oka Y., Sly W., Ryba N.J., Zuker C.S. (2009). The taste of carbonation. Science.

[38] Huang A.L., Chen X., Hoon M.A., Chandrashekar J., Guo W., Trankner D., Ryba N.J., Zuker C.S. (2006). The cells and logic for mammalian sour taste detection. Nature.

[39] Huang Y.A., Maruyama Y., Stimac R., Roper S.D. (2008). Presynaptic (type III) cells in mouse taste buds sense sour (acid) taste. J. Physiol..

[40] Tomchik S.M., Berg S., Kim J.W., Chaudhari N., Roper S.D. (2007). Breadth of tuning and taste coding in mammalian taste buds. J. Neurosci..

[41] Di Lorenzo P.M., Chen J.Y., Victor J.D. (2009). Quality time: Representation of a multidimensional sensory domain through temporal coding. J. Neurosci..

[42] Miller P., Katz D.B. (2010). Stochastic transitions between neural states in taste processing and decision-making. J. Neurosci..

[43] Jones L.M., Fontanini A., Katz D.B. (2006). Gustatory processing: A dynamic systems approach. Curr. Opin. Neurobiol..

[44] Fox A.L. (1932). The relationship between chemical constitution and taste. Proc. Natl. Acad. Sci. USA.

[45] Bartoshuk L.M. (2000). Comparing sensory experiences across individuals: Recent psychophysical advances illuminate genetic variation in taste perception. Chem. Senses..

[46] Bartoshuk L.M. (1993). The biological basis of food perception and acceptance. Food Qual. Pref..

[47] Bartoshuk L.M., Duffy V.B., Miller I.J. (1994). PTC/PROP tasting: Anatomy, psychophysics, and sex effects. Physiol. Behav..

[48] Guo S.W., Reed D.R. (2001). The genetics of phenylthiocarbamide perception. Ann. Hum. Biol..

[49] Bajec M.R., Pickering G.J. (2008). Thermal taste, PROP responsiveness, and perception of oral sensations. Physiol. Behav..

[50] Yeomans M.R., Tepper B.J., Rietzschel J., Prescott J. (2007). Human hedonic responses to sweetness: Role of taste genetics and anatomy. Physiol. Behav..

[51] Essick G.K., Chopra A., Guest S., McGlone F. (2003). Lingual tactile acuity, taste perception, and the density and diameter of fungiform papillae in female subjects. Physiol. Behav..

[52] Shahbake M., Hutchinson I., Laing D.G., Jinks A.L. (2005). Rapid quantitative assessment of fungiform papillae density in the human tongue. Brain Res..

[53] Melis M., Atzori E., Cabras S., Zonza A., Calo C., Muroni P., Nieddu M., Padiglia A., Sogos V., Tepper B.J. (2013). The gustin (CA6) gene polymorphism, rs2274333 (A/G), as a mechanistic link between PROP tasting and fungiform taste papilla density and maintenance. PLoS One.

[54] Tepper B.J., Nurse R.J. (1997). Fat perception is related to PROP taster status. Physiol. Behav..

[55] Kalmus H. (1958). Improvements in the classification of the taster genotypes. Ann. Hum. Genet..

[56] Bufe B., Breslin P.A., Kuhn C., Reed D.R., Tharp C.D., Slack J.P., Kim U.K., Drayna D., Meyerhof W. (2005). The molecular basis of individual differences in phenylthiocarbamide and propylthiouracil bitterness perception. Curr. Biol..

[57] Kim U.K., Jorgenson E., Coon H., Leppert M., Risch N., Drayna D. (2003). Positional cloning of the human quantitative trait locus underlying taste sensitivity to phenylthiocarbamide. Science.

[58] Hayes J.E., Bartoshuk L.M., Kidd J.R., Duffy V.B. (2008). Supertasting and PROP bitterness depends on more than the TAS2R38 gene. Chem. Senses..

[59] Lipchock S.V., Mennella J.A., Spielman A.I., Reed D.R. (2013). Human bitter perception correlates with bitter receptor messenger RNA expression in taste cells. Am. J. Clin. Nutr..

[60] Olson J.M., Boehnke M., Neiswanger K., Roche A.F., Siervogel R.M. (1989). Alternative genetic models for the inheritance of the phenylthiocarbamide taste deficiency. Genet. Epidemiol..

[61] Drayna D., Coon H., Kim U.K., Elsner T., Cromer K., Otterud B., Baird L., Peiffer A.P., Leppert M., Utah Genetic Reference Project (2003). Genetic analysis of a complex trait in the Utah Genetic Reference Project: A major locus for ptc taste ability on chromosome 7q and a secondary locus on chromosome 16p. Hum. Genet..

[62] Reed D.R., Nanthakumar E., North M., Bell C., Bartoshuk L.M., Price R.A. (1999). Localization of a gene for bitter-taste perception to human chromosome 5p15. Am. J. Hum. Genet..

[63] Matsuo R. (2000). Role of saliva in the maintenance of taste sensitivity. Crit. Rev. Oral. Biol. Med..

[64] Bennick A. (2002). Interaction of plant polyphenols with salivary proteins. Crit. Rev. Oral. Biol. Med..

[65] Dinnella C., Recchia A., Fia G., Bertuccioli M., Monteleone E. (2009). Saliva characteristics and individual sensitivity to phenolic astringent stimuli. Chem. Senses.

[66] Dinnella C., Recchia A., Tuorila H., Monteleone E. (2011). Individual astringency responsiveness affects the acceptance of phenol-rich foods. Appetite.

[67] Dinnella C., Recchia A., Vincenzi S., Tuorila H., Monteleone E. (2010). Temporary modification of salivary protein profile and individual responses to repeated phenolic astringent stimuli. Chem. Senses.

[68] Cabras T., Melis M., Castagnola M., Padiglia A., Tepper B.J., Messana I., Tomassini Barbarossa I. (2012). Responsiveness to 6-*n*-propylthiouracil (PROP) is associated with salivary levels of two specific basic proline-rich proteins in humans. PLoS One.

[69] Azen E.A., Latreille P., Niece R.L. (1993). PRBI gene variants coding for length and null polymorphisms among human salivary Ps, PmF, PmS, and Pe proline-rich proteins (PRPs). Am. J. Hum. Genet..

[70] Melis M., Aragoni M.C., Arca M., Cabras T., Caltagirone C., Castagnola M., Crnjar R., Messana I., Tepper B.J., Barbarossa I.T. (2013). Marked increase in PROP taste responsiveness following oral supplementation with selected salivary proteins or their related free amino acids. PLoS One.

[71] Padiglia A., Zonza A., Atzori E., Chillotti C., Calo C., Tepper B.J., Barbarossa I.T. (2010). Sensitivity to 6-*n*-propylthiouracil is associated with gustin (carbonic anhydrase VI) gene polymorphism, salivary zinc, and body mass index in humans. Am. J. Clin. Nutr..

[72] Henkin R.I., Martin B.M., Agarwal R.P. (1999). Efficacy of exogenous oral zinc in treatment of patients with carbonic anhydrase VI deficiency. Am. J. Med. Sci..

[73] Calo C., Padiglia A., Zonza A., Corrias L., Contu P., Tepper B.J., Barbarossa I.T. (2011). Polymorphisms in TAS2R38 and the taste bud trophic factor, gustin gene co-operate in modulating PROP taste phenotype. Physiol. Behav..

[74] Genick U.K., Kutalik Z., Ledda M., Destito M.C.S., Souza M.M., Cirillo C.A., Godinot N., Martin N., Morya E., Sameshima K. (2011). Sensitivity of genome-wide-association signals to phenotyping strategy: The PROP-TAS2R38 taste association as a benchmark. Plos One.

[75] Feeney E.L., Hayes J.E. (2014). Exploring associations between taste perception, oral anatomy and polymorphisms in the carbonic anhydrase (gustin) gene CA6. Physiol. Behav..

[76] Goldstein G.L., Daun H., Tepper B.J. (2007). Influence of PROP taster status and maternal variables on energy intake and body weight of pre-adolescents. Physiol. Behav..

[77] Whissell-Buechy D., Wills C. (1989). Male and female correlations for taster (P.T.C.) phenotypes and rate of adolescent development. Ann. Hum. Biol..

[78] Zuniga J.R., Davis S.H., Englehardt R.A., Miller I.J., Schiffrman S.S., Phillips C. (1993). Taste performance on the anterior human tongue varles with fungiform taste bud density. Chem. Senses.

[79] Correa M., Hutchinson I., Laing D.G., Jinks A.L. (2013). Changes in fungiform papillae density during development in humans. Chem. Senses.

[80] Gayathri Devi A., Henderson S.A., Drewnowski A. (1997). Sensory acceptance of Japanese green tea and soy products is linked to genetic sensitivity to 6-*n*-propylthiouracil. Nutr. Cancer.

[81] Bartoshuk L.M., Duffy V.B., Lucchina L.A., Prutkin J., Fast K. (1998). PROP (6-*n*-propylthiouracil) supertasters and the saltiness of NaCl. Ann. N. Y. Acad. Sci..

[82] Bartoshuk L., Fast K., Karrer T., Marino S., Price R., Reed D. (1992). PROP supertasters and the perception of sweetness and bitterness. Chem. Senses.

[83] Bartoshuk L.M. (1979). Bitter taste of saccharin related to the genetic ability to taste the bitter substance 6-*n*-propylthiouracil. Science.

[84] Bartoshuk L.M., Rifkin B., Marks L.E., Bars P. (1986). Taste and aging. J. Gerontol..

[85] Bartoshuk L.M., Rifkin B., Marks L.E., Hooper J.E. (1988). Bitterness of KCl and benzoate: Related to genetic status for sensitivity to PTC/PROP. Chem. Senses.

[86] Gent J., Bartoshuk L. (1983). Sweetness of sucrose, neohesperidin dihydrochalcone, and saccharin is related to genetic ability to taste the bitter substance 6-*n*-propylthiouracil. Chem. Senses.

[87] Prescott J., Swain-Campbell N. (2000). Responses to repeated oral irritation by capsaicin, cinnamaldehyde and ethanol in PROP tasters and non-tasters. Chem. Senses.

[88] Ly A., Drewnowski A. (2001). PROP (6-*n*-propylthiouracil) tasting and sensory responses to caffeine, sucrose, neohesperidin dihydrochalcone and chocolate. Chem. Senses.

[89] Duffy V.B., Davidson A.C., Kidd J.R., Kidd K.K., Speed W.C., Pakstis A.J., Reed D.R., Snyder D.J., Bartoshuk L.M. (2004). Bitter receptor gene (TAS2R38), 6-*n*-propylthiouracil (PROP) bitterness and alcohol intake. Alcohol. Clin. Exp. Res..

[90] Hayes J.E., Duffy V.B. (2007). Revisiting sugar-fat mixtures: Sweetness and creaminess vary with phenotypic markers of oral sensation. Chem. Senses.

[91] Duffy V.B., Bartoshuk L.M., Lucchina L.A., Snyder D.J., Tym A. (1996). Supertasters of PROP (6-*n*-propylthiouracil) rate the highest creaminess to high-fat milk products. Chem. Senses.

[92] Tepper B.J., Nurse R.J. (1998). PROP taster status is related to fat perception and preference. Ann. N. Y. Acad. Sci..

[93] Keller K.L., Steinmann L., Nurse R.J., Tepper B.J. (2002). Genetic taste sensitivity to 6-*n*-propylthiouracil influences food preference and reported intake in preschool children. Appetite.

[94] Dinehart M.E., Hayes J.E., Bartoshuk L.M., Lanier S.L., Duffy V.B. (2006). Bitter taste markers explain variability in vegetable sweetness, bitterness, and intake. Physiol. Behav..

[95] Tsuji M., Nakamura K., Tamai Y., Wada K., Sahashi Y., Watanabe K., Ohtsuchi S., Ando K., Nagata C. (2012). Relationship of intake of plant-based foods with 6-*n*-propylthiouracil sensitivity and food neophobia in Japanese preschool children. Eur. J. Clin. Nutr..

[96] Robino A., Mezzavilla M., Pirastu N., Dognini M., Tepper B.J., Gasparini P. (2014). A population-based approach to study the impact of PROP perception on food liking in populations along the silk road. PLoS One.

[97] Duffy V., Lucchina L., Bartoshuk L., Prescott J., Tepper B.J. (2004). Genetic variation in taste: Potential biomarker for cardiovascular disease risk?. Genetic Variations in Taste Sensitivity.

[98] Prescott J., Bartoshuk L.M., Prutkin J., Prescott J., Tepper B.J. (2004). 6-*n*-Propylthiouracil tasting and the perception of nontaste oral sensations. Genetic Variation in Taste Sensitivity.

[99] Hayes J.E., Duffy V.B. (2008). Oral sensory phenotype identifies level of sugar and fat required for maximal liking. Physiol. Behav..

[100] Kirkmeyer S.V., Tepper B.J. (2003). Understanding creaminess perception of dairy products using free-choice profiling and genetic responsivity to 6-*n*-propylthiouracil. Chem. Senses.

[101] Drewnowski A., Henderson S.A., Barratt-Fornell A. (1998). Genetic sensitivity to 6-*n*-propylthiouracil and sensory responses to sugar and fat mixtures. Physiol. Behav..

[102] Drewnowski A., Henderson S.A., Cockroft J.E. (2007). Genetic sensitivity to 6-*n*-propylthiouracil has no influence on dietary patterns, body mass indexes, or plasma lipid profiles of women. J. Am. Diet. Assoc..

[103] Duffy V.B., Bartoshuk L.M. (2000). Food acceptance and genetic variation in taste. J. Am. Diet. Assoc..

[104] Forrai G., Bankovi G. (1984). Taste perception for phenylthiocarbamide and food choice—A Hungarian twin study. Acta Physiol. Hung..

[105] Tepper B.J., Neilland M., Ullrich N.V., Koelliker Y., Belzer L.M. (2011). Greater energy intake from a buffet meal in lean, young women is associated with the 6-*n*-propylthiouracil (PROP) non-taster phenotype. Appetite.

[106] Tepper B.J., Ullrich N.V. (2002). Influence of genetic taste sensitivity to 6-*n*-propylthiouracil (PROP), dietary restraint and disinhibition on body mass index in middle-aged women. Physiol. Behav..

[107] Tepper B.J., Koelliker Y., Zhao L., Ullrich N.V., Lanzara C., D’Adamo P., Ferrara A., Ulivi S., Esposito L., Gasparini P. (2008). Variation in the bitter-taste receptor gene TAS2R38, and adiposity in a genetically isolated population in southern italy. Obesity.

[108] Shafaie Y., Koelliker Y., Hoffman D.J., Tepper B.J. (2013). Energy intake and diet selection during buffet consumption in women classified by the 6-*n*-propylthiouracil bitter taste phenotype. Am. J. Clin. Nutr..

[109] Gorovic N., Afzal S., Tjonneland A., Overvad K., Vogel U., Albrechtsen C., Poulsen H.E. (2011). Genetic variation in the HTAS2R38 taste receptor and *Brassica* vegetable intake. Scand. J. Clin. Lab. Invest..

[110] Feeney E., O’Brien S., Scannell A., Markey A., Gibney E.R. (2011). Genetic variation in taste perception: Does it have a role in healthy eating?. Proc. Nutr. Soc..

[111] Baranowski T., Baranowski J.C., Watson K.B., Jago R., Islam N., Beltran A., Martin S.J., Nguyen N., Tepper B.J. (2011). 6-*n*-Propylthiouracil taster status not related to reported cruciferous vegetable intake among ethnically diverse children. Nutr. Res..

[112] Mennella J.A., Pepino M.Y., Reed D.R. (2005). Genetic and environmental determinants of bitter perception and sweet preferences. Pediatrics.

[113] O’Brien S.A., Feeney E.L., Scannell A.G., Markey A., Gibney E.R. (2013). Bitter taste perception and dietary intake patterns in irish children. J. Nutrigenet. Nutrigenomics.

[114] Tepper B.J., Christensen C.M., Cao J. (2001). Development of brief methods to classify individuals by PROP taster status. Physiol. Behav..

[115] Zhao L., Kirkmeyer S.V., Tepper B.J. (2003). A paper screening test to assess genetic taste sensitivity to 6-*n*-propylthiouracil. Physiol. Behav..

[116] Mennella J., Pepino M.Y., Duke F., Reed D. (2010). Age modifies the genotype-phenotype relationship for the bitter receptor TAS2R38. BMC Genet..

[117] Timpson N., Heron J., Day I., Ring S., Bartoshuk L., Horwood J., Emmett P., Davey-Smith G. (2007). Refining associations between TAS2R38 diplotypes and the 6-*n*-propylthiouracil (PROP) taste test: Findings from the AVON Longitudinal Study of Parents and Children. BMC Genet..

[118] Running C.A., Mattes R.D., Tucker R.M. (2013). Fat taste in humans: Sources of within- and between-subject variability. Prog. Lipid Res..

[119] Than T.T., Delay E.R., Maier M.E. (1994). Sucrose threshold variation during the menstrual cycle. Physiol. Behav..

[120] Alberti-Fidanza A., Fruttini D., Servili M. (1998). Gustatory and food habit changes during the menstrual cycle. Int. J. Vitam. Nutr. Res..

[121] Glanville E.V., Kaplan A.R. (1965). Taste perception and the menstrual cycle. Nature.

[122] Kuga M., Ikeda M., Suzuki K. (1999). Gustatory changes associated with the menstrual cycle. Physiol. Behav..

[123] Whissell-Buechy D. (1990). Effects of age and sex on taste sensitivity to phenylthiocarbamide (PTC) in the berkeley guidance sample. Chem. Senses.

[124] Glanville E.V., Kaplan A.R., Fischer R. (1964). Age, sex, and taste sensitivity. J. Gerontol..

[125] Mojet J., Christ-Hazelhof E., Heidema J. (2001). Taste perception with age: Generic or specific losses in threshold sensitivity to the five basic tastes?. Chem. Senses.

[126] Stunkard A.J., Messick S. (1985). The three-factor eating questionnaire to measure dietary restraint, disinhibition and hunger. J. Psychosom. Res..

[127] Laessle R.G., Tuschl R.J., Kotthaus B.C., Pirke K.M. (1989). Behavioral and biological correlates of dietary restraint in normal life. Appetite.

[128] Tepper B.J., Trail A.C., Shaffer S.E. (1996). Diet and physical activity in restrained eaters. Appetite.

[129] Alexander J.M., Tepper B.J. (1995). Use of reduced-calorie/reduced-fat foods by young adults: Influence of gender and restraint. Appetite.

[130] Westenhoefer J. (1991). Dietary restraint and disinhibition: Is restraint a homogeneous construct?. Appetite.

[131] Lawson O.J., Williamson D.A., Champagne C.M., DeLany J.P., Brooks E.R., Howat P.M., Wozniak P.J., Bray G.A., Ryan D.H. (1995). The association of body weight, dietary intake, and energy expenditure with dietary restraint and disinhibition. Obes. Res..

[132] Bryant E.J., King N.A., Blundell J.E. (2008). Disinhibition: Its effects on appetite and weight regulation. Obes. Rev..

[133] Tomassini Barbarossa I., Carta G., Murru E., Melis M., Zonza A., Vacca C., Muroni P., di Marzo V., Banni S. (2013). Taste sensitivity to 6-*n*-propylthiouracil is associated with endocannabinoid plasma levels in normal-weight individuals. Nutrition.

[134] Silvestri C., di Marzo V. (2013). The endocannabinoid system in energy homeostasis and the etiopathology of metabolic disorders. Cell Metab..

[135] Banni S., di Marzo V. (2010). Effect of dietary fat on endocannabinoids and related mediators: Consequences on energy homeostasis, inflammation and mood. Mol. Nutr. Food Res..

[136] Piscitelli F., Carta G., Bisogno T., Murru E., Cordeddu L., Berge K., Tandy S., Cohn J.S., Griinari M., Banni S. (2011). Effect of dietary krill oil supplementation on the endocannabinoidome of metabolically relevant tissues from high-fat-fed mice. Nutr. Metab..

[137] DiPatrizio N.V., Astarita G., Schwartz G., Li X., Piomelli D. (2011). Endocannabinoid signal in the gut controls dietary fat intake. Proc. Natl. Acad. Sci. USA.

[138] Monteleone P., Piscitelli F., Scognamiglio P., Monteleone A.M., Canestrelli B., di Marzo V., Maj M. (2012). Hedonic eating is associated with increased peripheral levels of ghrelin and the endocannabinoid 2-arachidonoyl-glycerol in healthy humans: A pilot study. J. Clin. Endocrinol. Metab..

[139] Mattes R.D. (2012). Spices and energy balance. Physiol. Behav..

[140] Mattes R.D. (2005). Fat taste and lipid metabolism in humans. Physiol. Behav..

[141] Astrup A., Andersen T., Christensen N.J., Bulow J., Madsen J., Breum L., Quaade F. (1990). Impaired glucose-induced thermogenesis and arterial norepinephrine response persist after weight reduction in obese humans. Am. J. Clin. Nutr..

